# Reaction-based Indicator displacement Assay (RIA) for the selective colorimetric and fluorometric detection of peroxynitrite†
†Electronic supplementary information (ESI) available. See DOI: 10.1039/c4sc03983a



**DOI:** 10.1039/c4sc03983a

**Published:** 2015-03-06

**Authors:** Xiaolong Sun, Karel Lacina, Elena C. Ramsamy, Stephen E. Flower, John S. Fossey, Xuhong Qian, Eric V. Anslyn, Steven D. Bull, Tony D. James

**Affiliations:** a Department of Chemistry , University of Bath , Bath , BA2 7AY , UK . Email: t.d.james@bath.ac.uk ; Email: s.d.bull@bath.ac.uk; b School of Chemistry , University of Birmingham , Edgbaston , Birmingham , West Midlands , B15 2TT , UK; c School of Pharmacy , East China University of Science and Technology , Meilong Road 130 , Shanghai 200237 , China; d Department of Chemistry and Biochemistry , The University of Texas at Austin , Austin , Texas 78712 , USA . Email: anslyn@austin.utexas.edu; e CEITEC , Masaryk University , Kamenice 5 , 62500 , Brno , Czech Republic

## Abstract

Using the self-assembly of aromatic boronic acids with Alizarin Red S (ARS), we developed a new chemosensor for the selective detection of peroxynitrite. Phenylboronic acid (PBA), benzoboroxole (BBA) and 2-(*N*,*N*-dimethylaminomethyl)phenylboronic acid (NBA) were employed to bind with ARS to form the complex probes. In particular, the ARS–NBA system with a high binding affinity can preferably react with peroxynitrite over hydrogen peroxide and other ROS/RNS due to the protection of the boron *via* the solvent-insertion B–N interaction. Our simple system produces a visible colorimetric change and on–off fluorescence response towards peroxynitrite. By coupling a chemical reaction that leads to an indicator displacement, we have developed a new sensing strategy, referred to herein as RIA (Reaction-based Indicator displacement Assay).

## Abstract 2

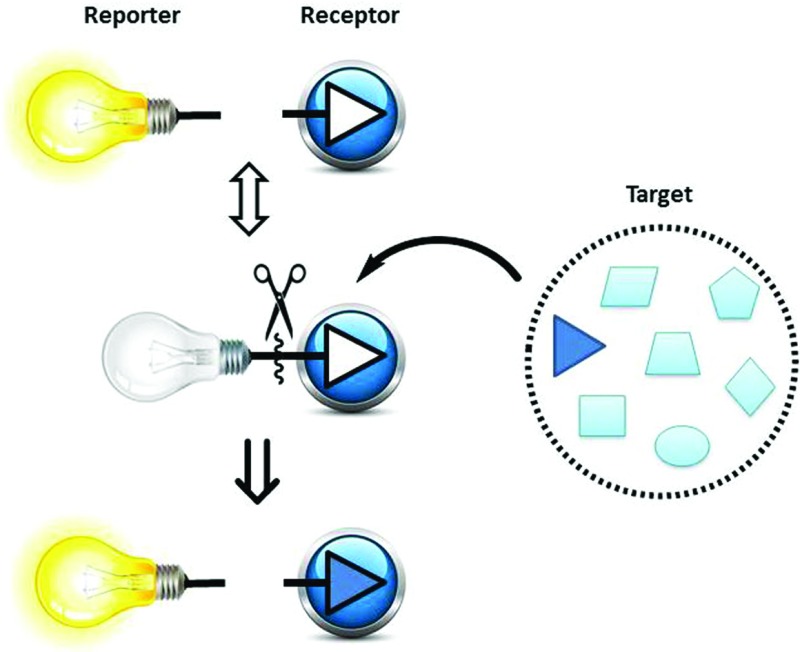
Using the self-assembly of aromatic boronic acids with Alizarin Red S (ARS), we developed a new chemosensor for the selective detection of peroxynitrite.

## Introduction

Peroxynitrite (ONOO^–^) – a combination of nitric oxide (NO˙) and the superoxide radical anion (O_2_^–^˙) – was first discovered as a biological endogenous oxidant in 1990.^1^ It is recognised as a strong oxidant with a very short lifetime (∼10 ms) in physiological and pathological processes. Peroxynitrite is a highly reactive molecule involved in cell signal transduction, and leads to apoptosis in HL-60 cells, and PC-12 cells. Many biomolecules are oxidised and/or nitrated by peroxynitrite-derived radicals, including DNA, tyrosine residues, thiols, and unsaturated fatty-acid-containing phospholipids. Endogenous peroxynitrite formation, and associated protein nitration, has been implicated in Alzheimer's disease, Parkinson's disease, Huntington's disease, amyotrophic lateral sclerosis, viral myocarditis, septic shock, cardiac allograft, transplant coronary artery disease, idiopathic dilated cardiomyopathy, atrial fibrillation, hypercholesterolemia, atherosclerosis, hypertension, diabetes, diabetic nephropathy, and traumatic brain injury.^2,3^ Thus, the importance of peroxynitrite has captured the attention of many groups who seek effective and selective approaches for its detection.

**Scheme 1:**
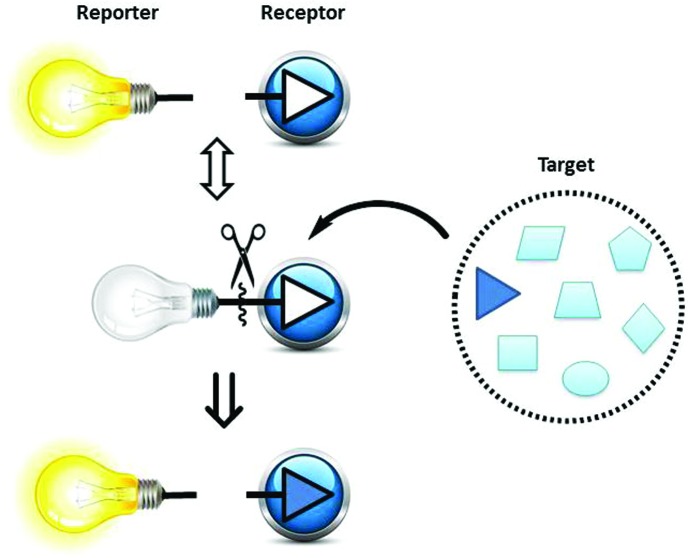
Schematic illustration of the proposed RIA: “reaction-based indicator displacement assay”. Firstly: the receptor and reporter interact reversibly. Secondly: the receptor and reporter are selectively cleaved by a chemical reaction triggered by the target. The change of the output signal for the RIA system can then be used to detect the target. (Graphic prepared using: Light Bulbs © Kraska/Shutterstock; Scissors © Mallinka1/Shutterstock and Blue Button © Roman Sotola/Shutterstock.)

Among the powerful tools available for ONOO^–^ detection are small-molecule probes which are attractive owing to their high sensitivity, easy manipulation, coupled with widely available instrumentation. To the best of our knowledge, limited work has been carried out in the development of small-molecule probes for the selective colorimetric sensing of peroxynitrite.^4,5^ The successful application of indicator displacement arrays, by Anslyn,^6–11^ Severin,^12^ Singaram,^13–18^ and reaction-based small-molecular fluorescent probes for chemoselective ROS detection, developed by Chang,^19,20^ inspired us to combine these two approaches and develop RIA: “Reaction-based Indicator displacement Assay” for the selective detection of peroxynitrite (ONOO^–^) (Scheme 1).

**Fig. 1:**
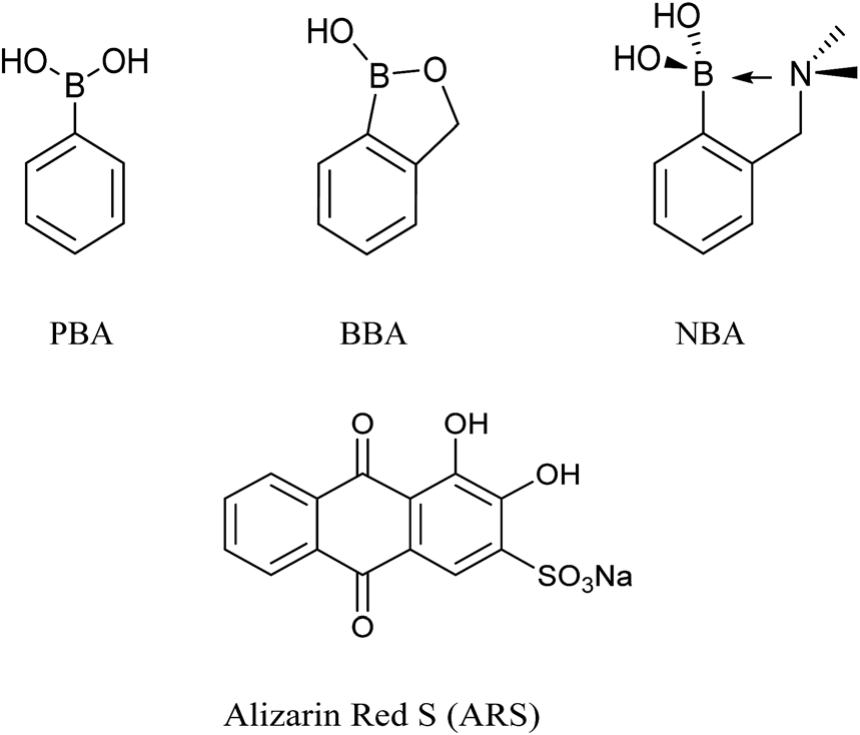
Structures of the various boronic acid receptors, phenylboronic acid (PBA), benzoboroxole (BBA), and 2-(*N*,*N*-dimethylaminomethyl)phenylboronic acid (NBA), as well as common indicator Alizarin Red S (ARS).

Phenylboronic acid (PBA, p*K*_a_ = 8.83), benzoboroxole (BBA, p*K*_a_ = 7.30), and 2-(*N*,*N*-dimethylaminomethyl)phenylboronic acid (NBA, p*K*_a_ = 6.70) are three representative receptors among common boronic acid motifs (Fig. 1), which have been extensively applied in the area of monosaccharides sensing,^21–23^ while, Alizarin Red S (ARS), has been successfully employed as a general optical reporter for investigating the binding of boronic acids with carbohydrates (Fig. 1).^23,24^ Also, some of our previous research has used the binding and analyte-mediated release of Alizarin Red S from hydrogel-bound boronic acids.^25^

Bearing this previous work in mind, we decided to evaluate the sensing systems (ARS–BAs), which are formed by the conjugation of aromatic boronic acid receptors with Alizarin Red S *in situ*, for the selective detection of peroxynitrite.

## Results and discussion

When buffered at neutral pH, we found only small changes occur *i.e.* only a slight blue-shift was observed in the UV-Vis absorption and a very small fluorescence response due to binding of PBA (200 μM), BBA (200 μM), NBA (200 μM) with ARS (50 μM), respectively (FS1, FS2). Also, the almost identical pink colours of the solutions clearly indicate weak binding between ARS and three boronic acid receptors at pH 7.30 (FS2). Therefore, we decided to use a higher pH working environment to enhance the binding of boronic acid with the catechol unit of ARS.

**Fig. 2:**
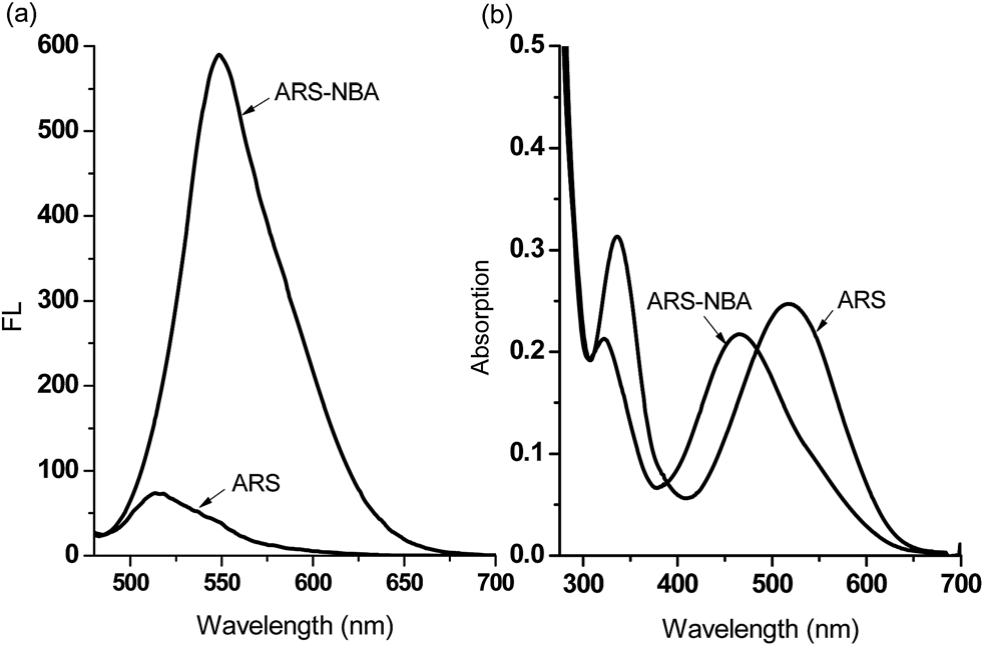
(a) Fluorescence spectra (*λ*_ex_ = 460 nm) and (b) absorption spectra for ARS only (50 μM), ARS–NBA (ARS, 50 μM; NBA, 200 μM). The complexes were formed *in situ*. The data was obtained in 52.1% MeOH/H_2_O PBS buffer (pH 8.10) at 25 °C.

**Scheme 2:**
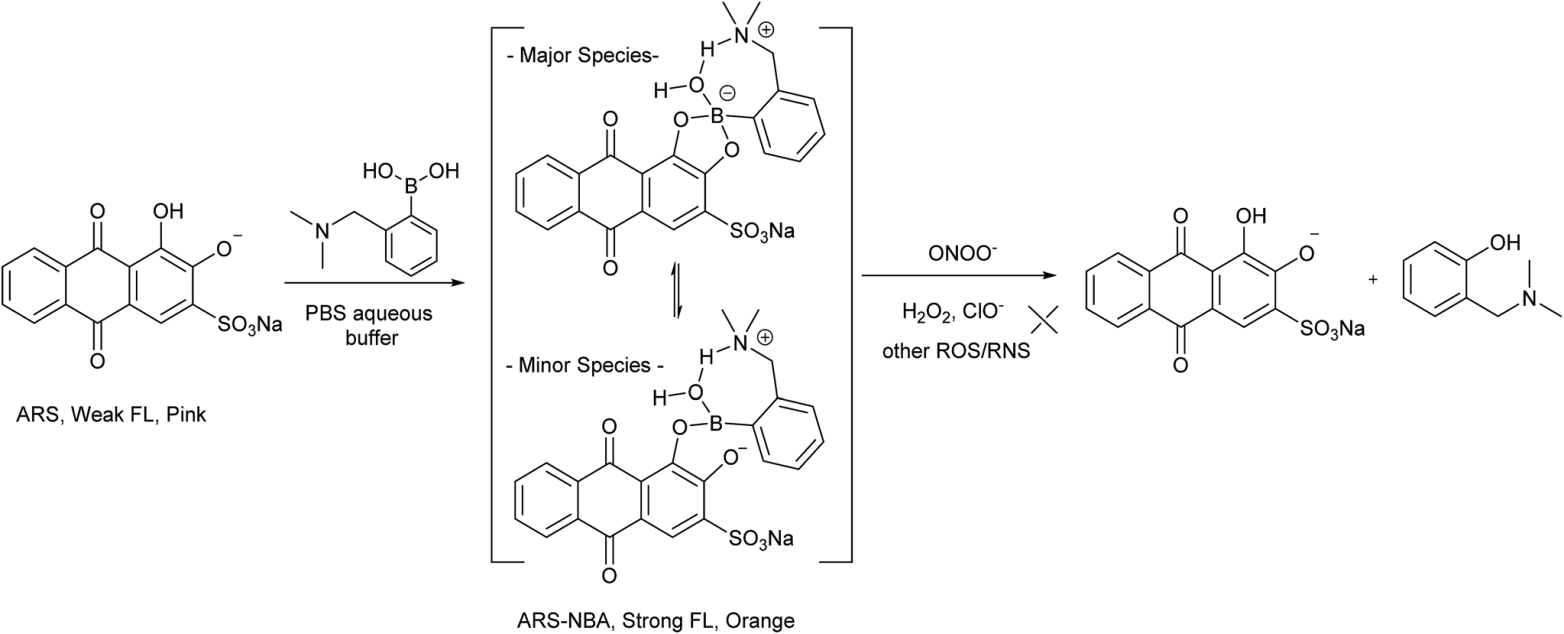
Sensing mechanism of ARS–NBA complex probe for peroxynitrite.

As can be seen from Fig. 2 in 52.1% MeOH/H_2_O PBS buffer (pH 8.10), compounds NBA, PBA and BBA behaved differently with ARS and in particular for the fluorescence, there is *ca.* 15.0-fold fluorescence increase for NBA (*F*/*F*_0_) while only a 3.4-fold increase is observed for PBA and a 2.8-fold increase with BBA (FS3), respectively. In terms of absorption, NBA (200 μM) produced the largest shift in wavelength from *λ*_max_ = 520 nm to *λ*_max_ = 465 nm (55 nm blue-shift) on binding with ARS, in comparison with a 10 nm blue-shift for BBA and a 20 nm blue-shift for PBA was observed. A significant colour change from pink to orange in the presence of NBA (200 μM) with ARS (50 μM) was observed (FS4), while BBA (200 μM) and PBA (200 μM) did not change significantly even after one night of stirring. Based upon recent results, the binding between NBA and ARS (binding constant *k* = 7200 ± 92 M^–1^, FS5), is most likely enhanced by the N–B interaction *via* a solvent insertion (Scheme 2). From the boron NMR (FS6), the NBA boron is clearly sp^3^ in nature and remains sp^3^ with added ARS under neutral and alkaline conditions (16 ppm) due to solvent insertion. The binding between ARS and NBA results in two species (one major and one minor with approximately 2 : 1 ratio) as described by Benkovic.^26^ (Scheme 2)

**Fig. 3:**
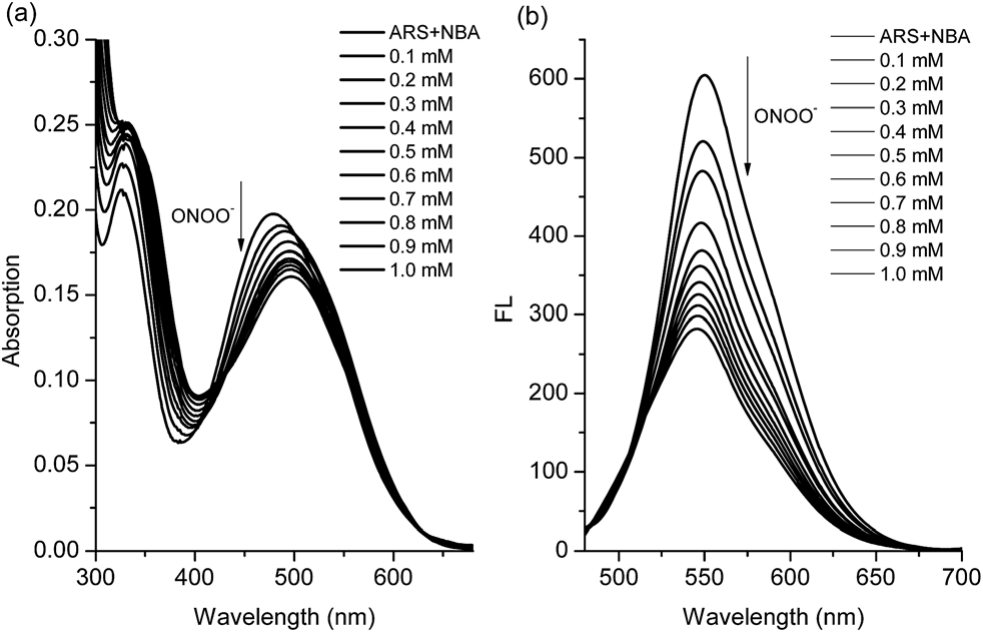
(a) UV-Vis titration and (b) fluorescence titration (*λ*_ex_ = 460 nm) for ARS–NBA (ARS, 50 μM; NBA, 200 μM) in the presence of various concentrations of ONOO^–^ (0–1 mM). The data was obtained in 52.1% MeOH/H_2_O PBS buffer (pH 8.10) at 25 °C.

We have previously demonstrated the N–B interaction provides protection for boronic acids towards common oxidants resulting in selectivity towards stronger reactive oxygen species such as peroxynitrite (ONOO^–^). To a preformed sensing ensemble, peroxynitrite (ONOO^–^) mediated oxidation of the aryl boronate ARS adduct led to phenols and release of ARS, thereby giving a fluorescence decrease and an accompanying red-shifted absorbance wavelength (Fig. 3).

As can be seen from the dose-dependent absorption response in Fig. 3a, when various concentrations of ONOO^–^ (0–1.0 mM) were added to a solution of ARS–NBA (ARS, 50 μM; NBA, 200 μM), a decrease in the absorption at *λ*_max_ = 465 nm was observed with the appearance of a red-shifted band centred at 500 nm (*A*_500nm_/*A*_465nm_ = 1.15). In the relationship between *A*_500nm_/*A*_465nm_ and concentration of peroxynitrite, there was rapid absorption increase in the presence of peroxynitrite below 0.4 mM while the reaction between ARS–NBA and ONOO^–^ (0.4–1.0 mM) was reduced due to saturation (FS7). In Fig. 3b, the fluorescence of the ARS–NBA complex was reduced to a *ca.* 0.45 of its original value (*F* (in the presence of ONOO^–^)/*F*_0_ (in the absence of ONOO^–^)) over a concentration range of ONOO^–^ from 0 to 1 mM. A linear relationship (*R* ^2^ = 0.994) was observed between the relative fluorescence ((*F* – *F*_0_)/*F* at *λ*_550nm_) and the peroxynitrite (ONOO^–^) concentration (0.4–1.0 mM, FS8).

Furthermore, due to the very short lifetime (∼10 ms) and low steady-state concentration (nM range) of peroxynitrite, we monitored the time dependant response towards peroxynitrite (using both UV-Vis and Fluorescence methods). From these time-drive experiments the chemical reaction between probe ARS–NBA and peroxynitrite proceeds rapidly (*k*′ = 4.39 s^–1^, *t*_1/2_ = 0.14 s, *k*_2_ = 8.78 × 10^4^ s^–1^ M^–1^, FS9 and FS10). Using dose-dependent titrations, we obtained a LOD (limit of detection) of 5.4 μM for the sensing of peroxynitrite (FS8). Importantly, these results indicate that the ARS–NBA system with an N–B interaction produce significant colorimetric and fluorometric response towards peroxynitrite.

As reported previously, hydrogen peroxide (H_2_O_2_), hypochlorite (ClO^–^) and peroxynitrite (ONOO^–^) react with boronate-based compounds to produce the phenol analogues.^27,28^ We have previously demonstrated that the solvent-insertion interaction between the amine and boron provides a protection for the boronic acid towards oxidation and results in the selective detection of peroxynitrite (ONOO^–^) over hydrogen peroxide (H_2_O_2_) and hypochlorite (ClO^–^) and other ROS/RNS species.^29,30^ Noticeably, we also observed that H_2_O_2_ is much more reactive with increasing pH. However, when we use NBA (2-(*N*,*N*-dimethylaminomethyl)phenylboronic acid) with ARS this provides a complex with a strong N–B interaction resulting in the selective sensing of peroxynitrite (ONOO^–^) over hydrogen peroxide (H_2_O_2_) and other ROS/RNS species.

**Fig. 4:**
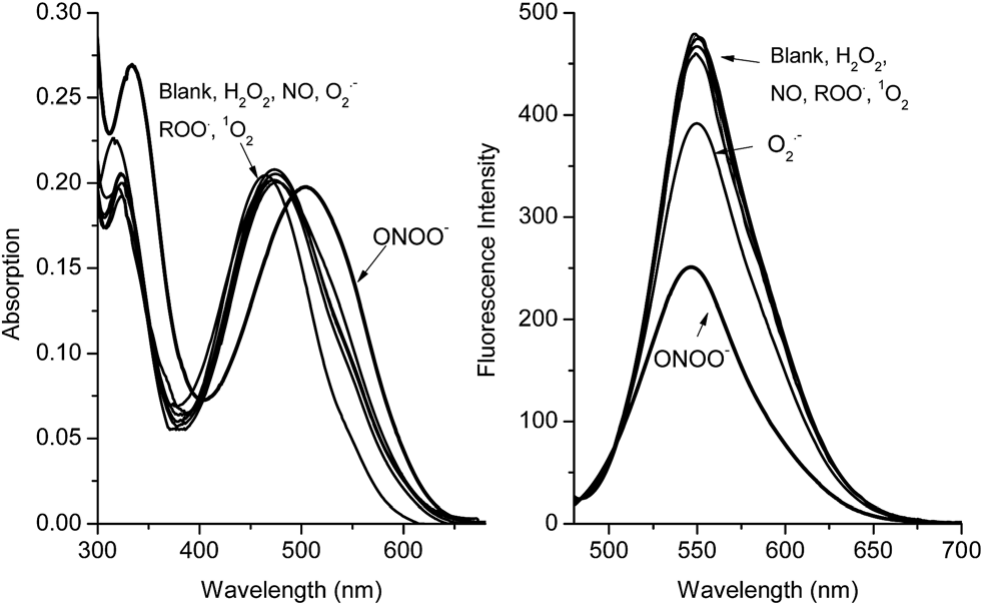
(a) Absorption spectra and (b) fluorescence spectra (*λ*_ex_ = 460 nm) for ARS–NBA (ARS, 50 μM; NBA, 200 μM) in the presence of blank, H_2_O_2_ (0.5 mM), NO (0.5 mM), O_2_^–^˙ (0.5 mM), AAPH (0.5 mM), ^1^O_2_ (0.5 mM), ONOO^–^ (0.5 mM) for 60 min. The data was obtained in 52.1% MeOH/H_2_O PBS buffer (pH 8.10) at 25 °C.

Both the absorption and emission spectra (Fig. 4) clearly demonstrate that the RIA system has excellent selectivity for peroxynitrite (ONOO^–^) over other common ROS. Additionally, high concentrations of H_2_O_2_ (1 mM) were not able to change the fluorescence and absorption intensity of the ARS–NBA complex (ARS, 50 μM; NBA, 200 μM) over 60 min (FS11), indicating, that the oxidation–reduction between boron and H_2_O_2_ has been prevented by the N–B interaction. The response of the ARS–NBA sensing system to hydroxyl HO˙ (500 μM), hypochlorite ClO^–^ (500 μM), and peroxynitrite ONOO^–^ (500 μM) (FS12) indicate that peroxynitrite reacts significantly more ((*F* – *F*_0_)/*F* = *ca.* 0.45) than with hydroxyl (*ca.* 0.29) and hypochlorite ClO^–^ (*ca.* 0.35). Importantly, both, hydroxyl HO˙ (500 μM) and hypochlorite ClO^–^ (500 μM) respond less than peroxynitrite ONOO^–^ (500 μM) in the RIA system FS12. However, hypochlorite (100 μM) can quench the fluorescence and change the absorption of just the ARS dye (FS13). Since hydroxyl is produced from the Fenton reaction (Fe^2+^ + H_2_O_2_), and is inherently coloured. Therefore, while both hydroxyl HO˙, hypochlorite ClO^–^ are not ideal ROS to evaluate the RIA colorimetric system, the system remains peroxynitrite selective (FS12). While, the other, ROS/RNS (NO, ^1^O_2_, ROO˙, O_2_^–^˙) (Fig. 4) do not change the absorption and emission spectra of the system significantly (FS14). Thus, the ARS–NBA sensing system can be used to selectively and sensitively detect peroxynitrite over other ROS/RNS in buffered media.

## Experimental

### Solvents and reagents

Solvents and reagents were reagent grade unless stated otherwise and were purchased from Fisher Scientific UK, Frontier Scientific Europe Ltd, TCI UK, Alfa Aesar and Sigma-Aldrich Company Ltd and were used without further purification.

### Fluorescence measurements

Fluorescence measurements were performed on a Perkin-Elmer Luminescence Spectrophotometer LS 50B and Gilden Photonics Ltd. FluoroSENS, utilising Starna Silica (quartz) cuvette with 10 mm path lengths, four faces polished. Data was collected *via* the Perkin-Elmer FL Winlab software package. All solvents used in fluorescence measurements were HPLC or fluorescence grade and the water was deionised.

### UV-Vis measurement

UV-Vis measurements were performed on a Perkin-Elmer Spectrophotometer. Absorption, utilising Starna Silica (quartz) cuvette with 10 mm path lengths, two faces polished. Data was collected *via* the Perkin-Elmer Lambda 20 software package. Further reprocess of the data was operated in OriginPro 8.0 graphing software.

### Preparation of ONOO^–^

Peroxynitrite stock solution was prepared by the reaction of hydrogen peroxide with sodium nitrite and stabilised in basic solution, which is frequently used for the *in vitro* experiments.^31^

Owing to its short-lived oxidant species, especially in the physiological pH (half-life, 10 ms), the preparation of peroxynitrite was carried out in the sodium hydroxide solution by chemical synthesis with a quenched flow reactor: using equal flow rates of the following solutions at 0 °C: 0.6 M KNO_2_, a solution 0.6 M in HCl and 0.7 M in H_2_O_2_, and 3 M NaOH.^31^ The product solution was analysed spectrophotometrically. The stock solution of peroxynitrite was fresh-made, and the pH of the stock solution (pH = 13.5) was adjusted by 12 N hydrochloric acid to pH = 12 and re-analysed each time in the detection experiments. The concentration of peroxynitrite was estimated by using an extinction coefficient of 1670 ± 50 cm^–1^ M^–1^ at 302 nm in 0.1 N sodium hydroxide (aq.).

### Preparation of ^–^OCl, H_2_O_2_ and other ROS/RNS

The concentration of ^–^OCl was determined from the absorption at 292 nm (*ε* = 350 M^–1^ cm^–1^);^32^ the concentration of H_2_O_2_ was determined from the absorption at 240 nm (*ε* = 43.6 M^–1^ cm^–1^); nitric oxide (NO) was prepared by treating a sodium nitrite solution (7.3 M) with sulfuric acid (3.6 M) and its stock solution (2.0 mM) was prepared by bubbling NO into deoxygenated deionised water for 30 min;^33^ ^1^O_2_ was generated by the reaction of H_2_O_2_ with NaClO; ROO˙ was generated from 2,2′-azobis(2-amidinopropane) dihydrochloride; superoxide was generated from KO_2_; hydroxyl radical was generated by Fenton reaction.

The UV-Vis spectra and fluorescent titrations with hydrogen peroxide were carried out at 25 °C in 1/15 M PBS buffer at pH 7.30 and 52.1% methanol/aqueous PBS buffer solution at pH 8.10.

## Conclusion

Using an indicator displacement assay induced by a chemical oxidation, we investigated a new chemo/biosensor for the selective sensing of peroxynitrite, using the self-assembly of boronic acid NBA with ARS as the reporter. The ARS–NBA system displays minimal response towards hydrogen peroxide (H_2_O_2_) and other ROS/RNS due to the protection provided by a B–N solvent-insertion interaction, but a large absorption and fluorescence response with peroxynitrite (ONOO^–^). The boronic acid receptor NBA is a good candidate for the selective detection of peroxynitrite as part of dye-displacement arrays and for biological applications, such as drug design and cell labelling experiments. The large initial change of fluorescence intensity indicates that the system might also be used as a temporal fluorescent probe to map intracellular ONOO^–^. Systems that produce colour changes are particular interesting since they could be incorporated into a diagnostic test paper for ONOO^–^, similar to universal indicator papers for pH. More importantly, ratiometric probes increase the dynamic range and permit signal-rationing, thus they provide a built-in correction for monitoring environmental effects.^34,35^ Therefore, we believe that this simple but powerful RIA system can be extended into new applications for the sensing of reactive oxygen and nitrogen species (ROS and RNS) both *in vitro* and *in vivo*.

## References

[cit1] Beckman J. S., Beckman T. W., Chen J., Marshall P. A., Freeman B. A. (1990). Proc. Natl. Acad. Sci. U. S. A..

[cit2] Pacher P., Beckman J. S., Liaudet L. (2007). Physiol. Rev..

[cit3] Szabo C., Ischiropoulos H., Radi R. (2007). Nat. Rev. Drug Discovery.

[cit4] Yang D., Wang H.-L., Sun Z.-N., Chung N.-W., Shen J.-G. (2006). J. Am. Chem. Soc..

[cit5] Zhang Q., Zhu Z., Zheng Y., Cheng J., Zhang N., Long Y. T., Zheng J., Qian X., Yang Y. (2012). J. Am. Chem. Soc..

[cit6] Wiskur S. L., Ait-Haddou H., Lavigne J. J., Anslyn E. V. (2001). Acc. Chem. Res..

[cit7] McCleskey S. C., Floriano P. N., Wiskur S. L., Anslyn E. V., McDevitt J. T. (2003). Tetrahedron.

[cit8] Goodey A., Lavigne J. J., Savoy S. M., Rodriguez M. D., Curey T., Tsao A., Simmons G., Wright J., Yoo S. J., Sohn Y., Anslyn E. V., Shear J. B., Neikirk D. P., McDevitt J. T. (2001). J. Am. Chem. Soc..

[cit9] Sohn Y. S., Goodey A., Anslyn E. V., McDevitt J. T., Shear J. B., Neikirk D. P. (2005). Biosens. Bioelectron..

[cit10] Umali A. P., Anslyn E. V. (2010). Curr. Opin. Chem. Biol..

[cit11] Nguyen B. T., Anslyn E. V. (2006). Coord. Chem. Rev..

[cit12] Buryak A., Severin K. (2004). Angew. Chem., Int. Ed..

[cit13] Schiller A., Vilozny B., Wessling R. A. and Singaram B., in Reviews in Fluorescence 2009, ed. C. D. Geddes, 2011, vol. 6, pp. 155–191.

[cit14] Cordes D., Miller A., Gamsey S., Singaram B. (2007). Anal. Bioanal. Chem..

[cit15] Schiller A., Wessling R. A., Singaram B. (2007). Angew. Chem., Int. Ed..

[cit16] Sharrett Z., Gamsey S., Fat J., Cunningham-Bryant D., Wessling R. A., Singaram B. (2007). Tetrahedron Lett..

[cit17] Camara J. N., Suri J. T., Cappuccio F. E., Wessling R. A., Singaram B. (2002). Tetrahedron Lett..

[cit18] Cordes D. B., Singaram B. (2012). Pure Appl. Chem..

[cit19] Lippert A. R., Van de Bittner G. C., Chang C. J. (2011). Acc. Chem. Res..

[cit20] Chan J., Dodani S. C., Chang C. J. (2012). Nat. Chem..

[cit21] Bull S. D., Davidson M. G., van den Elsen J. M., Fossey J. S., Jenkins A. T., Jiang Y. B., Kubo Y., Marken F., Sakurai K., Zhao J., James T. D. (2013). Acc. Chem. Res..

[cit22] Feng L., Wu L., Qu X. (2013). Adv. Mater..

[cit23] He F., Tang Y., Yu M., Wang S., Li Y., Zhu D. (2006). Adv. Funct. Mater..

[cit24] Springsteen G., Wang B. (2001). Chem. Commun..

[cit25] Ma W. M. J., Pereira Morais M. P., D'Hooge F., van den Elsen J. M. H., Cox J. P. L., James T. D., Fossey J. S. (2009). Chem. Commun..

[cit26] Tomsho J. W., Benkovic S. J. (2012). J. Org. Chem..

[cit27] Sikora A., Zielonka J., Lopez M., Joseph J., Kalyanaraman B. (2009). Free Radical Biol. Med..

[cit28] Zielonka J., Sikora A., Hardy M., Joseph J., Dranka B. P., Kalyanaraman B. (2012). Chem. Res. Toxicol..

[cit29] Sun X., Xu Q., Kim G., Flower S. E., Lowe J. P., Yoon J., Fossey J. S., Qian X., Bull S. D., James T. D. (2014). Chem. Sci..

[cit30] Sun X., Xu S. Y., Flower S. E., Fossey J. S., Qian X., James T. D. (2013). Chem. Commun..

[cit31] Reed J. W., Ho H. H., Jolly W. L. (1974). J. Am. Chem. Soc..

[cit32] Abo M., Urano Y., Hanaoka K., Terai T., Komatsu T., Nagano T. (2011). J. Am. Chem. Soc..

[cit33] Yang Y., Seidlits S. K., Adams M. M., Lynch V. M., Schmidt C. E., Anslyn E. V., Shear J. B. (2010). J. Am. Chem. Soc..

[cit34] Xu Z., Kim S. K., Han S. J., Lee C., Kociok-Kohn G., James T. D., Yoon J. (2009). Eur. J. Org. Chem..

[cit35] Kubo Y., Yamamoto M., Ikeda M., Takeuchi M., Shinkai S., Yamaguchi S., Tamao K. (2003). Angew. Chem., Int. Ed..

[cit36] Fossey J. S., Brittain W. D. G. (2015). Org. Chem. Front..

