# Supplementary material for: Reaction-based Indicator displacement Assay (RIA) for the selective colorimetric and fluorometric detection of peroxynitrite
Source: Chem Sci. 2015 Mar 6;6(5):2963–7. doi: 10.1039/c4sc03983a (PMC5490052; doi:10.1039/c4sc03983a)
Supplement: Supplementary file 1 [file SC-006-C4SC03983A-s001.pdf]

Electronic Supplementary Material (ESI) for Chemical Science.
This journal is © The Royal Society of Chemistry 2015

Supporting information

Reaction-based Indicator displacement Assay (RIA) for the selective colorimetric and fluorimetric detection of peroxynitrite†

Xiaolong Sun,[a] Karel Lacina,[a,e] Elena C. Ramsamy,[a] Stephen E. Flower,[a] John S. Fossey,[b] Xuhong Qian,[c] Eric V. Anslyn[d*] Steven D. Bull[a*] and Tony D. James[a*]

[a] *Department of Chemistry, University of Bath, BA2 7AY, UK;*

[b] *School of Chemistry, University of Birmingham, Edgbaston, Birmingham, B15 2TT, UK;*

[c] *School of Pharmacy, East China University of Science and Technology, Meilong Road 130, Shanghai 200237, China;*

[d] *Department of Chemistry and Biochemistry, The University of Texas at Austin, Austin, Texas, 78712, United States;*

[e] *CEITEC, Masaryk University, Kamenice 5, 62500, Brno, Czech Republic.*

Contents

1. **Supplementary spectra** 2
2. **Notes and references** 7
3. **NMR spectra** 7

Supporting information

1. Supplementary spectra

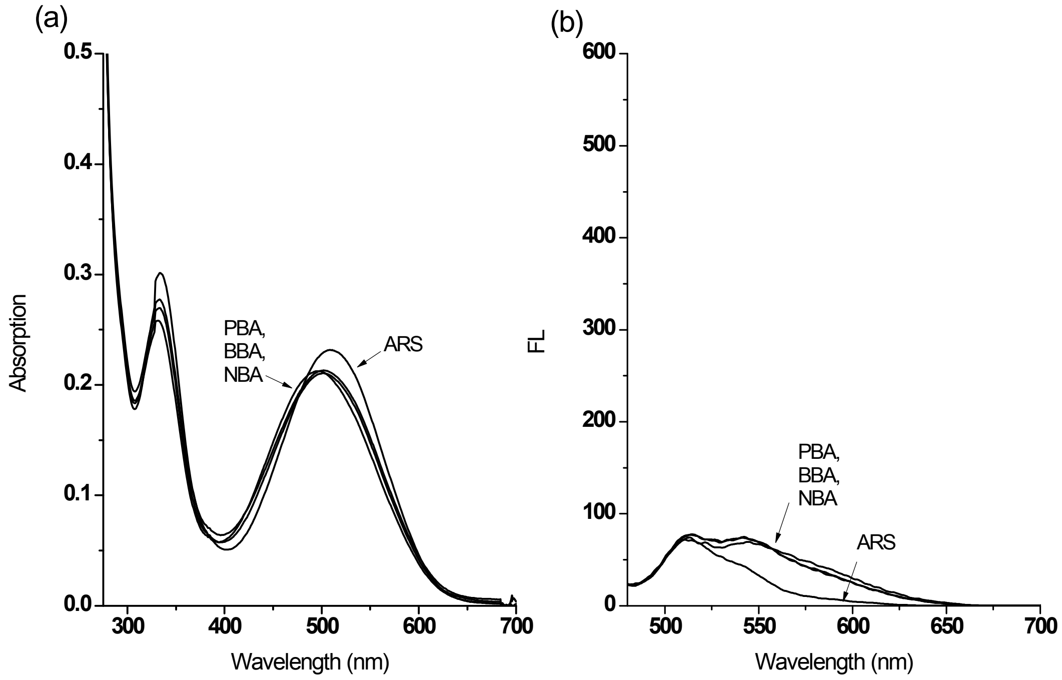

Figure S1. *(a) UV-Vis absorption spectra and (b) Fluorescence spectra (λ_{ex} = 460 nm) of ARS only (50 µM), ARS-PBA (ARS, 50 µM; PBA, 200 µM), ARS-BBA (ARS, 50 µM; BBA, 200 µM), ARS-NBA (ARS, 50 µM; NBA, 200 µM). The data was obtained in 1/15 M PBS buffer (pH 7.30) solution at 25 °C.*

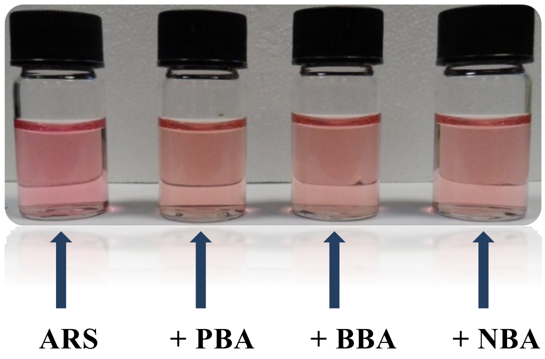

Figure S2. *Color images for ARS (50 µM), ARS-PBA (ARS, 50 µM; PBA, 200 µM), ARS-BBA (ARS, 50 µM; BBA, 200 µM) and ARS-NBA (ARS, 50 µM; NBA, 200 µM). The pictures were taken in 1/15 M PBS buffer (pH 7.30) at 25 °C.*

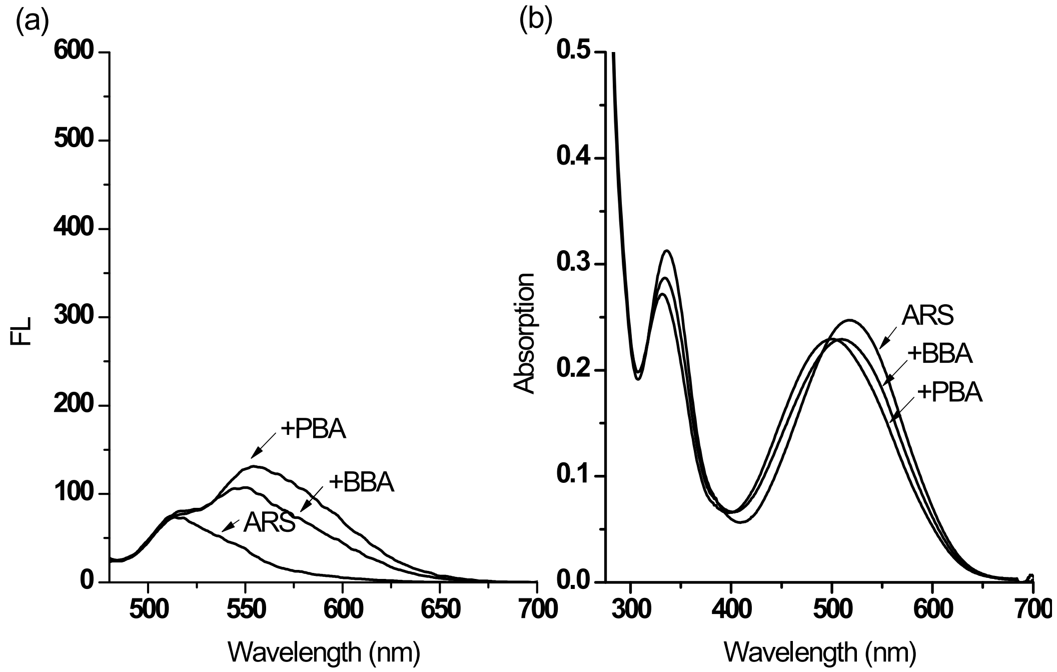

Figure S3. *(a) Fluorescence spectra (λ_{ex} = 460 nm) and (b) Absorption spectra for ARS only (50 µM), ARS-PBA (ARS, 50 µM; PBA, 200 µM), ARS-BBA (ARS, 50 µM; BBA, 200 µM). The complexes were formed in situ. The data was obtained in 52.1% MeOH/H_2O PBS buffer (pH 8.10) at 25 °C.*

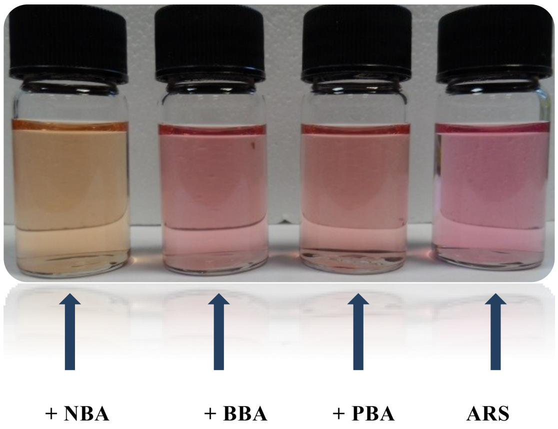

Figure S4. *Color images for ARS-NBA (ARS, 50 µM; NBA, 200 µM), ARS-BBA (ARS, 50 µM; BBA, 200 µM), ARS-PBA (ARS, 50 µM; PBA, 200 µM), ARS (50 µM). The pictures were taken in 52.1% MeOH/H_2O PBS buffer（pH 8.10）at 25 °C.*

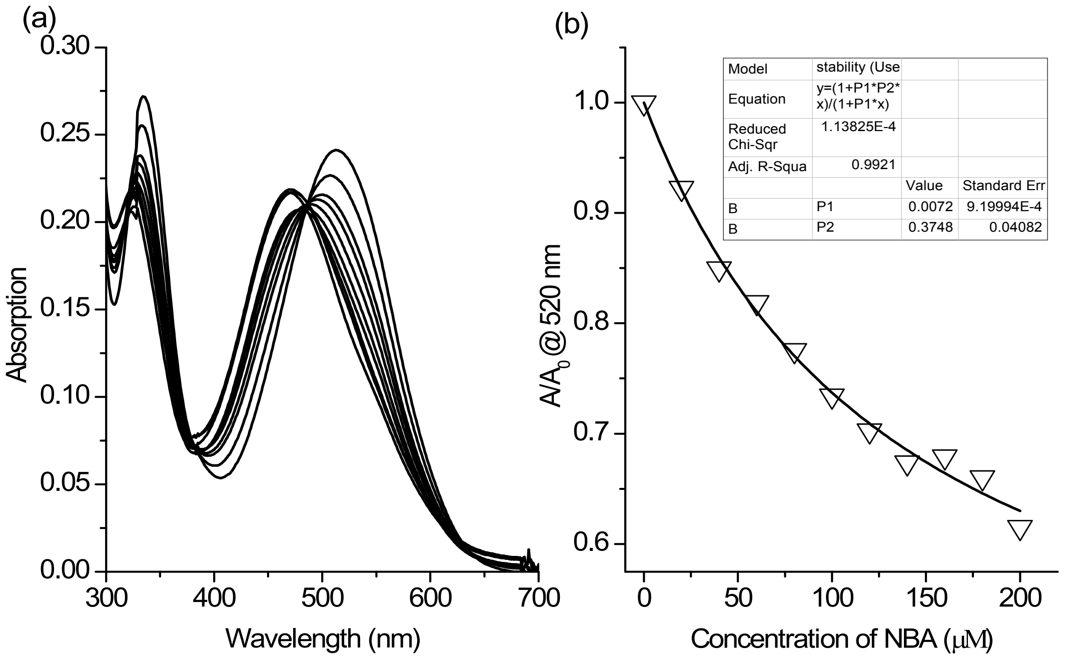

Figure S5. *(a) UV-Vis absorption titration spectra of ARS (50 μM) and addition of various concentrations of NBA (0 – 200 μM). (b) Curve fitting and binding constant calculation between ARS and NBA. The data were taken in 52.1% MeOH/H_2O PBS buffer（pH 8.10）at 25 °C.*

Binding constant calculation:

$$Y = (1 + kY_{lim}X)/(1 + kX) \qquad \textbf{Equation 1}$$

By fitting the relationship curve between absorption intensity and concentration of NBA using equation 1, we resulted:

$$k = 7200 \pm 92\ M^{-1}$$

$$Y_{lim} = 0.37 \pm 0.04$$

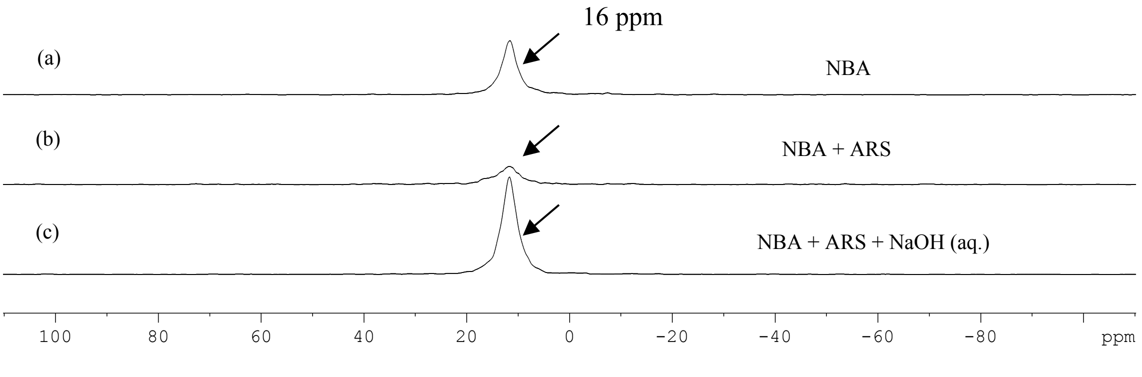

Figure S6. *^{11}B NMR for (a) NBA (10 mM); (b) in the presence of ARS (10 mM); (c) drop addition of NaOH (10 N) in MeOD/D_2O = 1:4.*

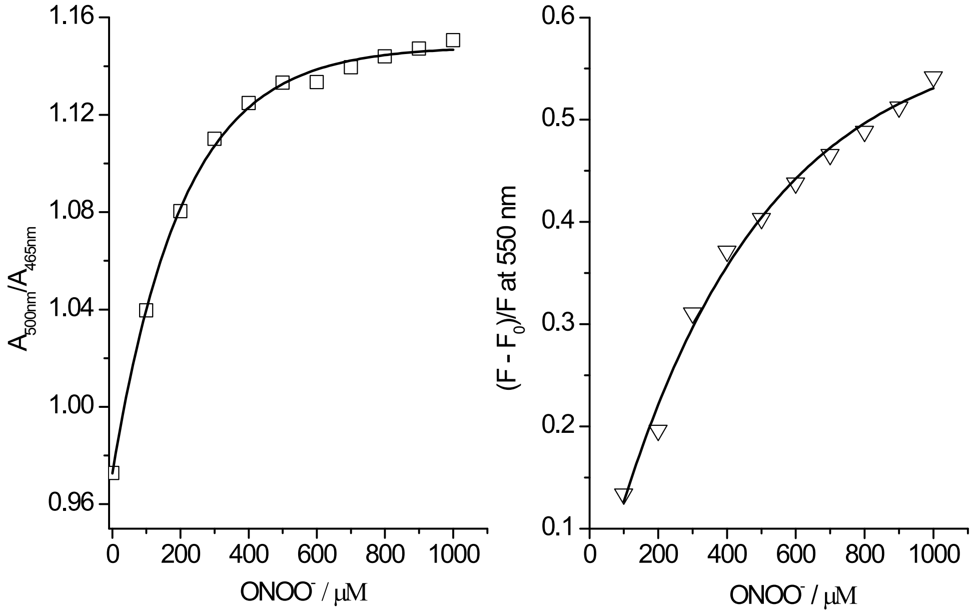

Figure S7. *(a) Relationship between A_{500nm}/A_{465nm} and concentration of $ONOO^-$ in UV-Vis titration and (b) Relationship between $(F - F_0)/F$ at λ_{550nm} and concentration of $ONOO^-$ in fluorescence titration for ARS-NBA (ARS, 50 μM; NBA, 200 μM) in the presence of various concentrations of $ONOO^-$ (0, 100 μM, 200 μM, 300 μM, 400 μM, 500 μM, 600 μM, 700 μM, 800 μM, 900 μM, 1000 μM). The data were taken in 52.1% MeOH/H_2O PBS buffer（pH 8.10）at 25 °C.*

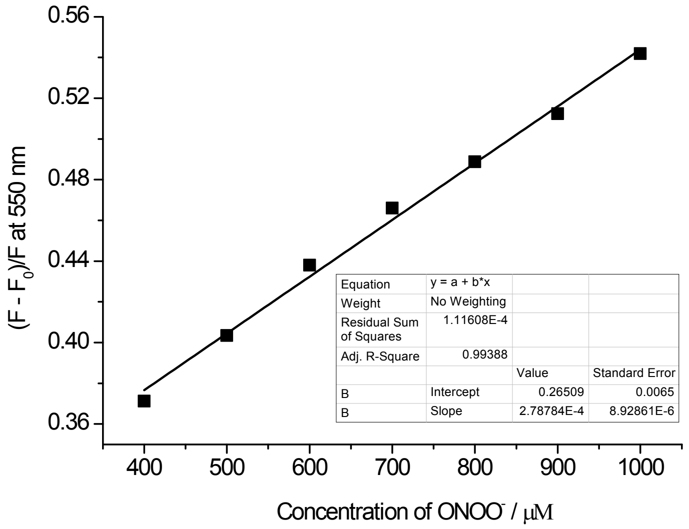

Figure S8. Linear *relationship between $(F - F_0)/F$ at λ_{550nm} and concentration of $ONOO^-$ in fluorescence titration for ARS-NBA (ARS, 50 μM; NBA, 200 μM) in the presence of various concentrations of $ONOO^-$ (0, 100 μM, 200 μM, 300 μM, 400 μM, 500 μM, 600 μM, 700 μM, 800 μM, 900 μM, 1000 μM). The data were taken in 52.1% MeOH/H_2O PBS buffer（pH 8.10）at 25 °C*

The detection limit can be calculated from the slope (k) and the standard deviation (σ) of the linearity curve.

LOD = $(3\times\sigma)/k$,

where σ = [Σ(y – y')*2/(n – 2)]/2;

k = slope of the linearity curve obtained by regression analysis;

n = number of points;

y = experimental response;

y' = calculated response;

By using the UV-Vis data, we calculated the LOD ($3\sigma/k$) = 5.4 μM.

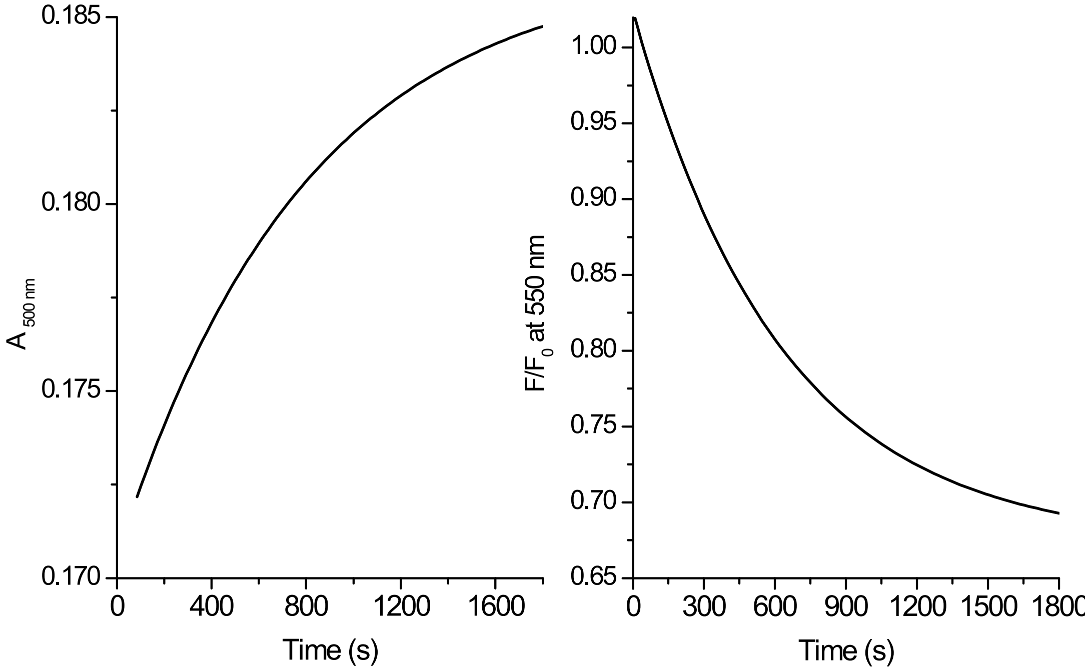

Figure S9. *Time dependant response for probe ARS-NBA (ARS, 50 µM; NBA, 200 µM) complex (a) UV-Vis Absorption at λ_{max} = 500 nm and (b) Fluorescence ratio change F/F_0 at λ_{max} = 550 nm in the presence of peroxynitrite (0.5 mM). The data were recorded in 52.1% MeOH/H_2O PBS buffer（pH 8.10）at 25 °C.*

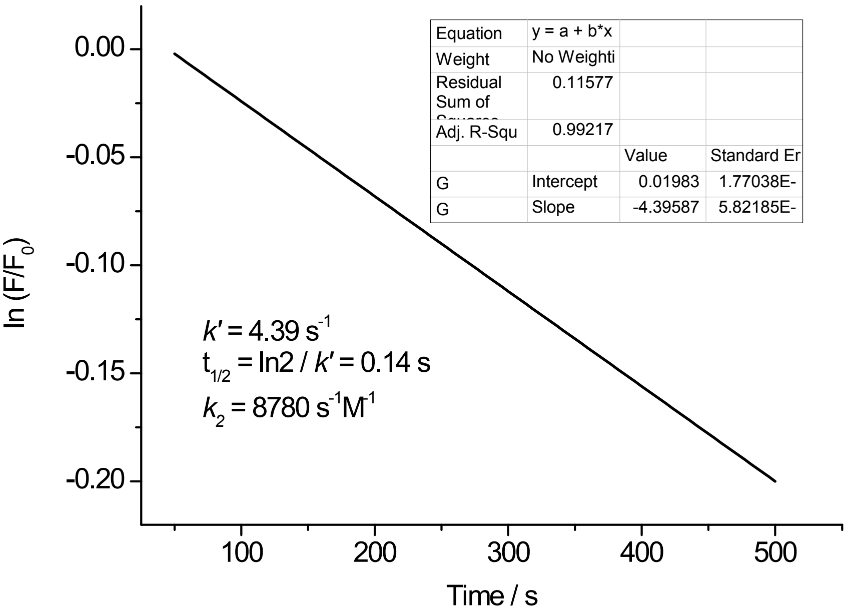

Figure S10. *Reaction rate constant calculation between fluorescence intensity $\ln(F/F_0)$ and time (0 – 500 seconds) for probe ARS-NBA (ARS, 50 µM; NBA, 200 µM) complex in the presence of peroxynitrite (0.5 mM).*

The reaction rate constant can be obtained through processing the data following a simple 1st order rate equation;

$F/F_0 = \exp(-(k't))$

We observed that a good linear relationship formed between $\ln(F/F_0)$ and time (0 – 500 seconds), hence,

It is calculated the k' = 4.39 s^{-1} from the slope.

Considering rate = k_2 [A][B] where A = concentration of ARS-NBA (50 µM), and B = concentration of $ONOO^-$ (500 µM), [B] >>

[A],

Therefore, rate = k'[A], where $k' = k_2$ [B],

We obtained $k_2 = 4.39\ s^{-1} / 0.5\ mM = 8.78 \times 10^3\ s^{-1}M^{-1}$

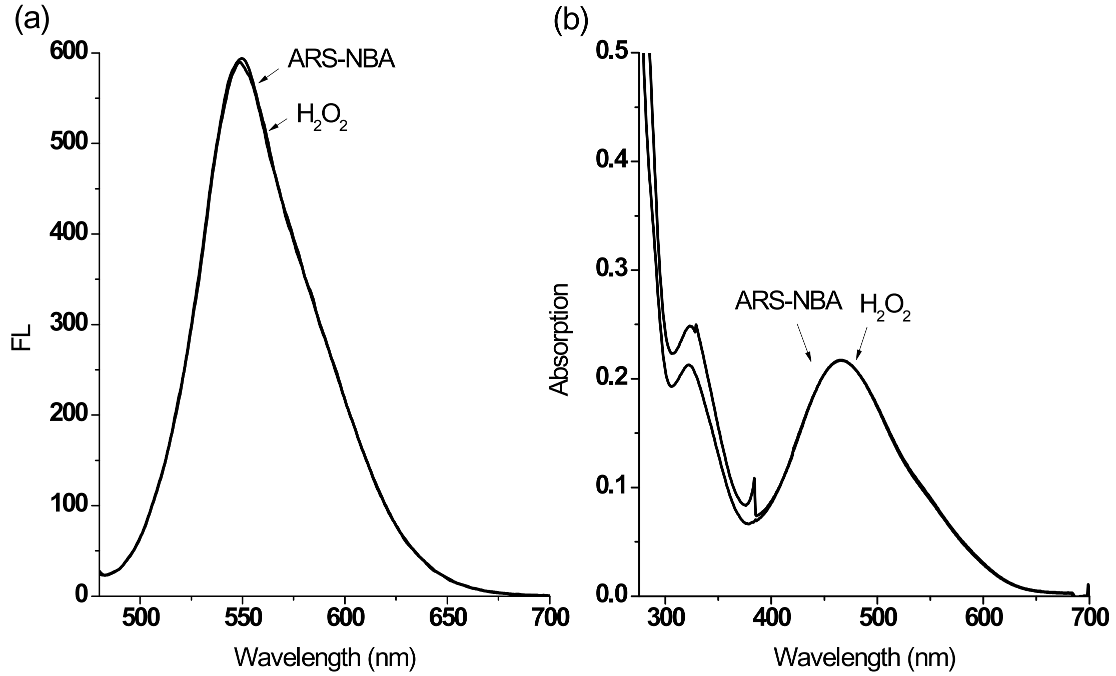

Figure S11. *(a) Fluorescence spectra (λ_{ex} = 460 nm) and (b) Absorption spectra for ARS-NBA (ARS, 50 μM; NBA, 200 μM) in the presence of H_2O_2 (1 mM) for 60 min. The data was obtained in 52.1% MeOH/H_2O PBS buffer (pH 8.10) at 25 °C.*

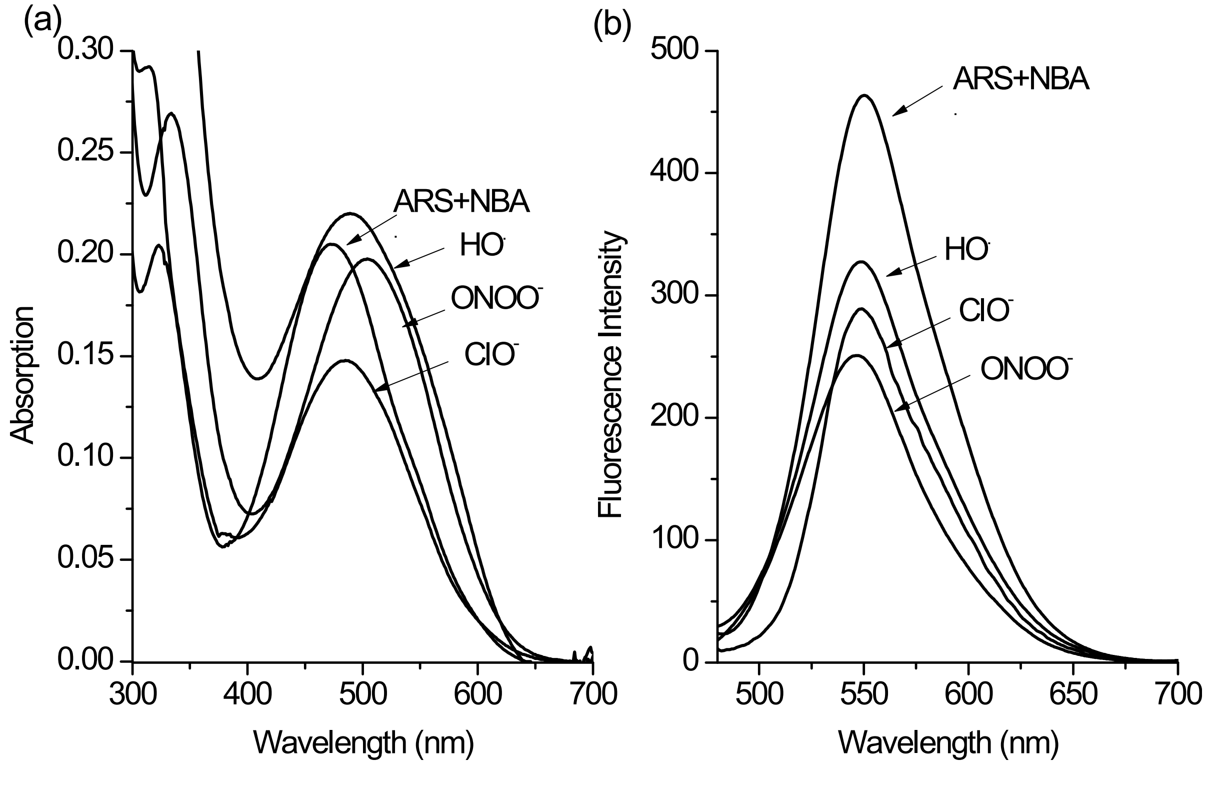

Figure S12. *(a) Absorption and (b) Fluorescence response of ARS-NBA (ARS, 50 μM; NBA, 200 μM) complex towards hydroxyl (0.5*

mM), hypochlorite (0.5 mM) and peroxynitrite (0.5 mM). The data was obtained in 52.1% MeOH/H_2O PBS buffer (pH 8.10) at 25 °C.

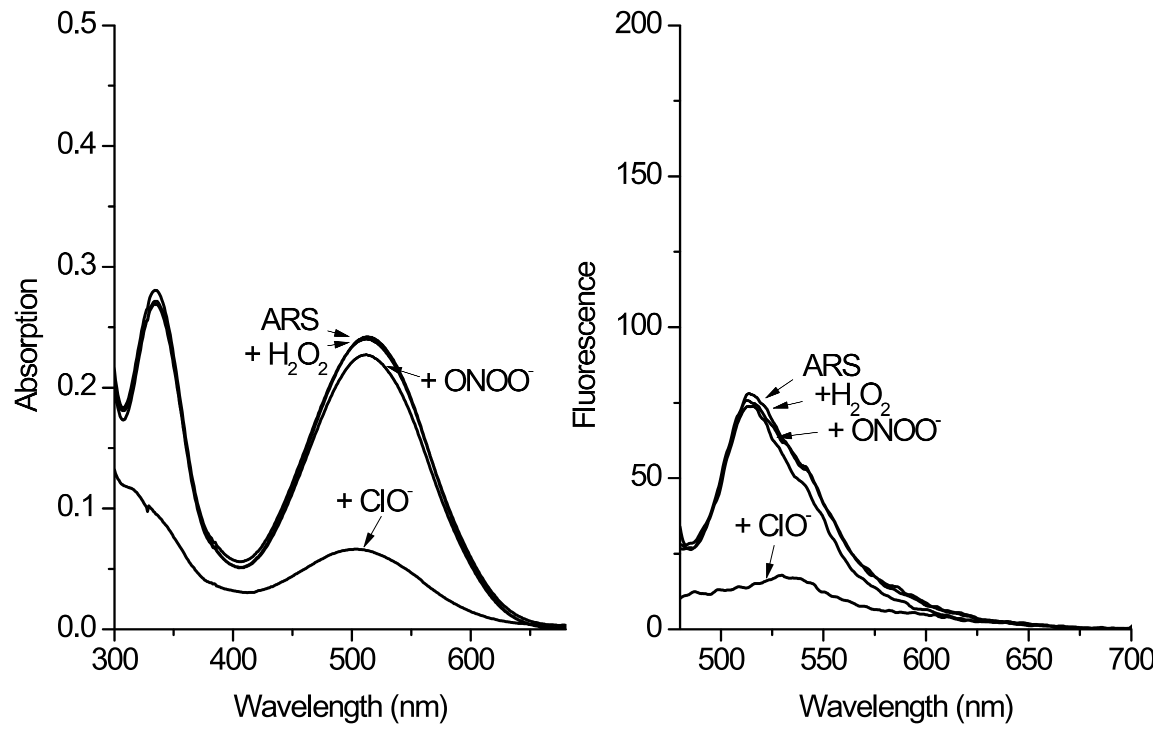

Figure S13. *(a) Absorption and (b) Fluorescence response of ARS (50 μM) complex towards H_2O_2 (0.1 mM), hypochlorite (0.1 mM), $ONOO^-$ (0.1 mM) for 60 min. The data was obtained in 52.1% MeOH/H_2O PBS buffer (pH 8.10) at 25 °C.*

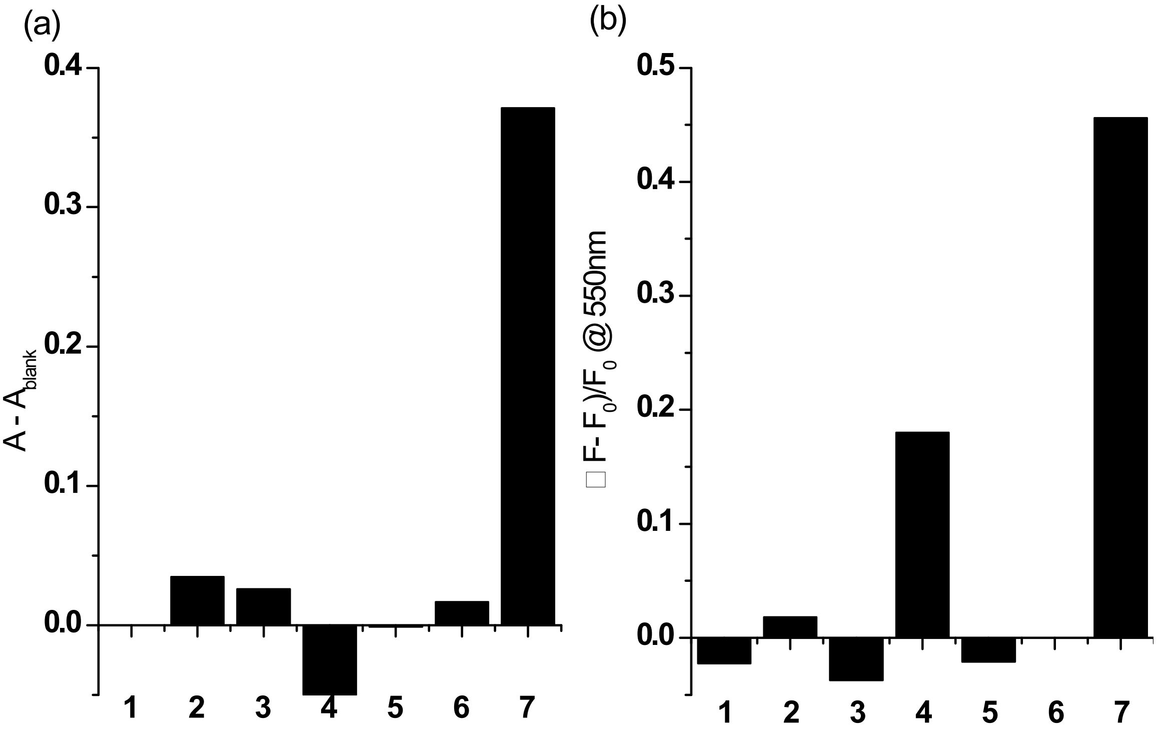

Figure S14. *Selectivity test of probe ARS-NBA (ARS, 50 μM; NBA, 200 μM) complex towards various ROS/RNS species. (a) column of UV-Vis absorption intensity A_{500nm}/A_{465nm}; (b) Column of fluorescence intensity $(F – F_0)/F_0$ at 550 nm in the presence of blank (1), H_2O_2 (2, 0.5 mM), NO (3, 0.5 mM), $O_2^{\cdot -}$ (4, 0.5 mM), AAPH (5, 0.5 mM), $^1O_2$ (6, 0.5 mM), $ONOO^-$ (7, 0.5 mM) for 60 min. The data*

was obtained in 52.1% MeOH/H_2O PBS buffer (pH 8.10) at 25 °C.

2. Notes and references

(1). J. W. Reed, H. H. Ho and W. L. Jolly, *J. Am. Chem. Soc.*, 1974, **96**, 1248.
(2). M. Abo, Y. Urano, K. Hanaoka, T. Terai, T. Komatsu and T. Nagano, *J. Am. Chem. Soc.*, 2011, **133**, 10629.

3. NMR spectra

Nuclear magnetic resonance (NMR) spectra were obtained in methanol-*D*. Where a Bruker AVANCE 300 was used, ^{1}H spectra were recorded at 300 MHz, ^{11}B spectra at 96 MHz and ^{13}C at 75 MHz. Chemical shifts (δ) are expressed in parts per million and are reported relative to the residual solvent peak as an internal standard in ^{1}H and ^{13}C spectra. The multiplicities and general assignments of the spectroscopic data are denoted as: singlet (s), doublet (d), unresolved multiplet (m), and broad (br).

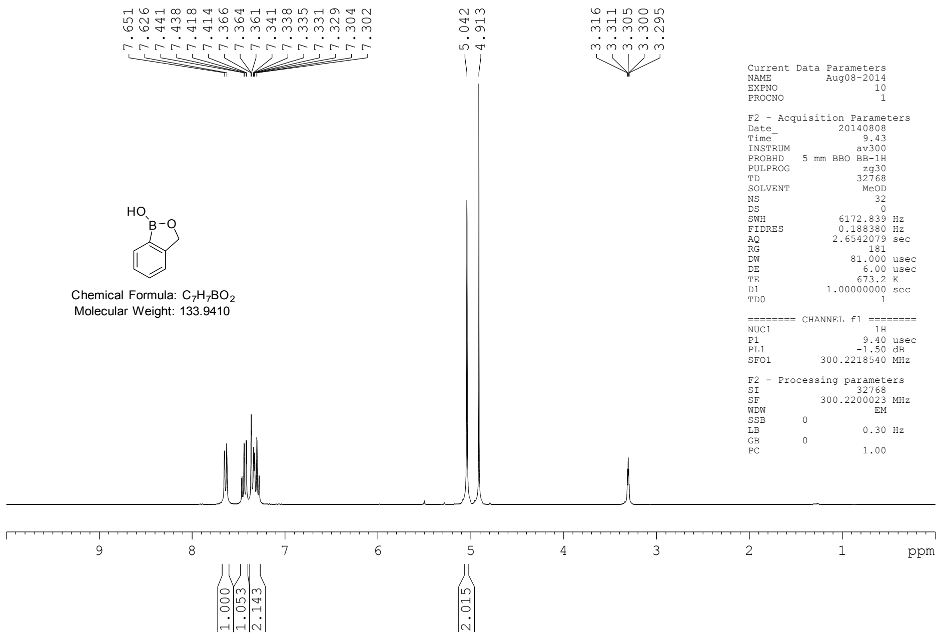

Figure S15. *^{1}H NMR of compound BBA in MeOD.*

Supporting information

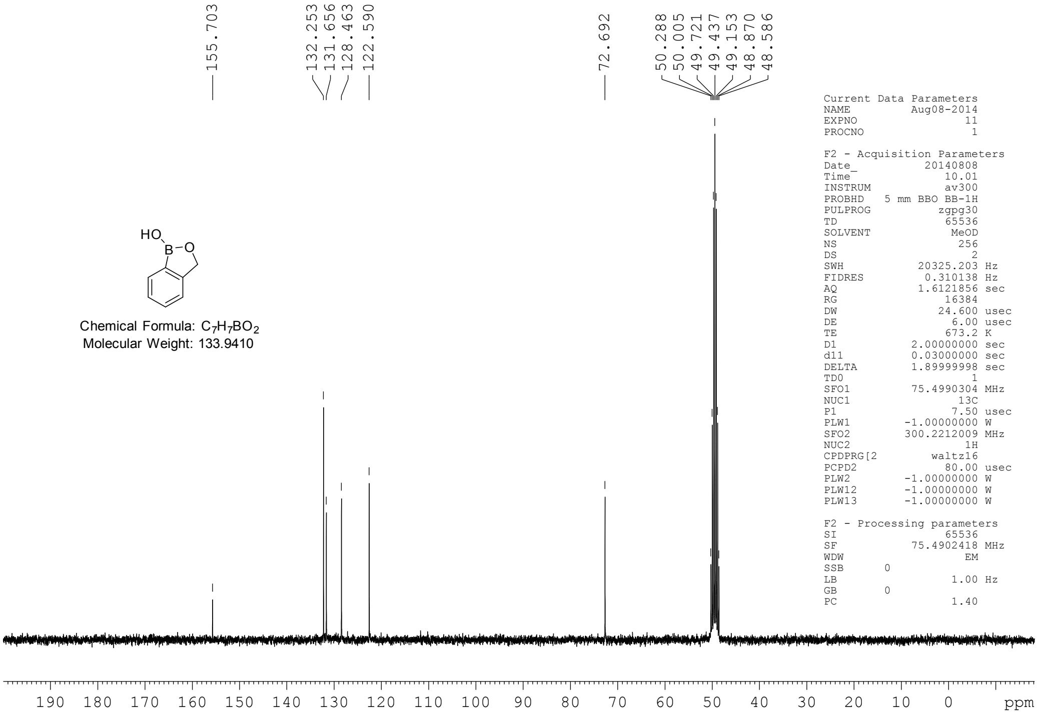

Figure S16. *^{13}C NMR of compound BBA in MeOD.*

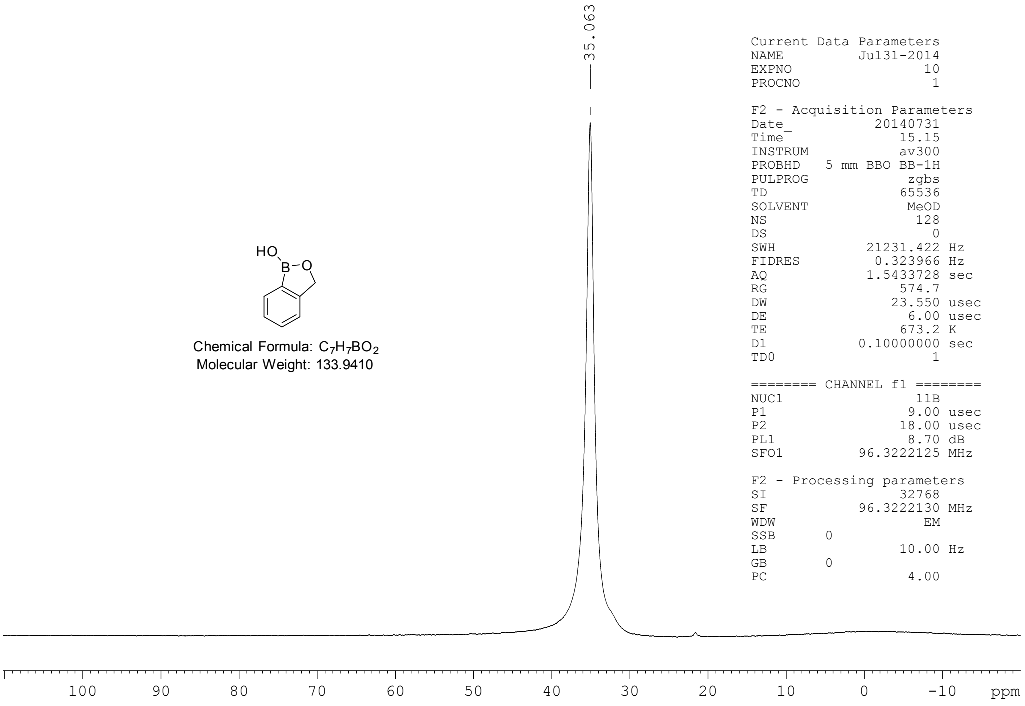

Figure S17. *^{11}B NMR of compound BBA in MeOD.*
